# Iodido(*N*-phenyl­thio­urea)bis­(triphenyl­phosphine)copper(I)

**DOI:** 10.1107/S1600536808019119

**Published:** 2008-06-28

**Authors:** Ruthairat Nimthong, Chaveng Pakawatchai, Saowanit Saithong, Jonathan P. H. Charmant

**Affiliations:** a Department of Chemistry, Faculty of Science, Prince of Songkla University, Hat Yai 90112, Thailand; b The School of Chemistry, University of Bristol, Cantock’s Close, Bristol BS 1TS, England

## Abstract

The coordination geometry of the Cu atom in the title compound, [CuI(C_7_H_8_N_2_S)(C_18_H_15_P)_2_], is distorted tetra­hedral; it is coordinated by two triphenyl­phosphine P atoms, one S atom from *N*-phenyl­thio­urea (ptu) and one I atom. The crystal structure is stabilized by intra- and inter­molecular N—H⋯I and N—H⋯S inter­actions.

## Related literature

For related literature, see: Aslanidis *et al.* (1993 ▶, 1998 ▶); Bowmaker *et al.* (1987 ▶); Cox *et al.* (1999 ▶); Jianping & Kazuyuki (1996 ▶); Karagiannidis *et al.* (1990 ▶); Lecomte *et al.* (1989 ▶); Skoulika *et al.* (1991 ▶).
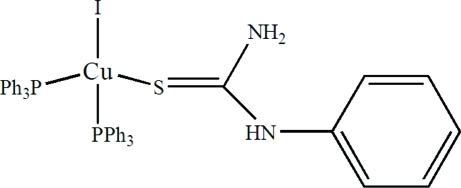


## Experimental

### 

#### Crystal data

[CuI(C_7_H_8_N_2_S)(C_18_H_15_P)_2_]
*M* *_r_* = 867.19
Triclinic, 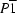

*a* = 10.9505 (9) Å
*b* = 18.7294 (15) Å
*c* = 21.3731 (18) Å
α = 67.422 (1)°
β = 77.215 (1)°
γ = 73.224 (1)°
*V* = 3844.9 (5) Å^3^
*Z* = 4
Mo *K*α radiation
μ = 1.54 mm^−1^
*T* = 173 (2) K
0.50 × 0.30 × 0.07 mm

#### Data collection

Bruker SMART Platform diffractometer
Absorption correction: multi-scan (*SADABS*; Bruker, 2003 ▶) *T*_min_ = 0.582, *T*_max_ = 0.898
42789 measured reflections
15698 independent reflections
8887 reflections with *I* > 2σ(*I*)
*R*_int_ = 0.088

#### Refinement

*R*[*F* ^2^ > 2σ(*F* ^2^)] = 0.050
*wR*(*F* ^2^) = 0.108
*S* = 0.98
15698 reflections
901 parameters
H-atom parameters constrained
Δρ_max_ = 0.82 e Å^−3^
Δρ_min_ = −0.74 e Å^−3^

### 

Data collection: *SMART* (Bruker, 1998 ▶); cell refinement: *SAINT* (Bruker, 2003 ▶); data reduction: *SAINT* and *SHELXTL* (Sheldrick, 2008 ▶); program(s) used to solve structure: *SHELXS97* (Sheldrick, 2008 ▶); program(s) used to refine structure: *SHELXL97* (Sheldrick, 2008 ▶); molecular graphics: *SHELXTL*; software used to prepare material for publication: *SHELXTL*.

## Supplementary Material

Crystal structure: contains datablocks I, global. DOI: 10.1107/S1600536808019119/hg2408sup1.cif

Structure factors: contains datablocks I. DOI: 10.1107/S1600536808019119/hg2408Isup2.hkl

Additional supplementary materials:  crystallographic information; 3D view; checkCIF report

## Figures and Tables

**Table 1:** Hydrogen-bond geometry (Å, °)

*D*—H⋯*A*	*D*—H	H⋯*A*	*D*⋯*A*	*D*—H⋯*A*
N1—H1*B*⋯S2^i^	0.88	2.58	3.455 (5)	173
N3—H3*B*⋯S1^ii^	0.88	2.63	3.396 (5)	146
N2—H2*A*⋯I1	0.88	2.65	3.511 (5)	166
N4—H4*A*⋯I2	0.88	2.71	3.567 (4)	165

Symmetry codes: (i) 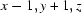; (ii) 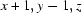.

**Fig. 1.**
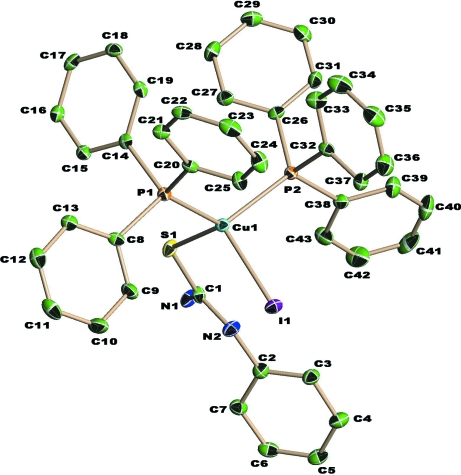
The molecular structure of the molecule A of [CuI(PPh3)2(ptu)] complex. Thermal ellipsoids are shown at 30% probability level.

**Fig. 2.**
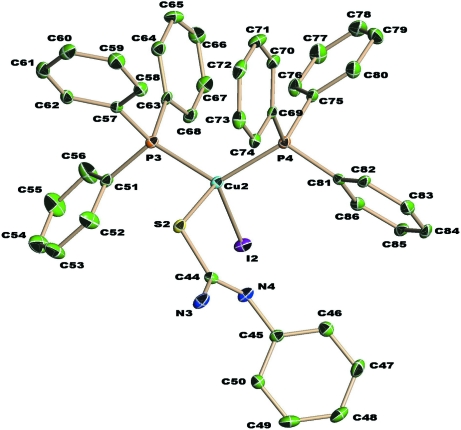
The molecular structure of the molecule B of [CuI(PPh3)2(ptu)] complex. Thermal ellipsoids are shown at 30% probability level.

**Fig. 3.**
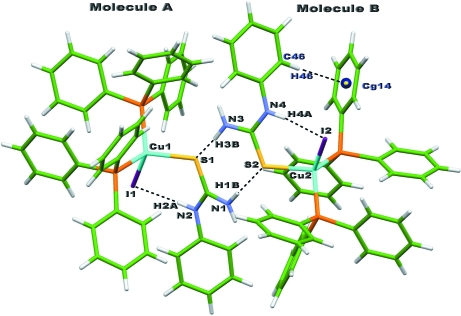
The intra- and inter-molecular interactions of [CuI(PPh3)2(ptu)] complex.

## supplementary crystallographic information

### Comment

Studies of copper(I) complexes with mixed ligand systems containing triphenylphosphine and ligands containing S and N donors, have been increasing because of the versatility of these ligands as well as the different steric characteristics of the phosphine ligands, which can modify the compound geometry (Cox *et al.*, 1999). The title complex, (I), is a monomeric complex which crystallizes in the triclinic system space group *P*1. The structure consists of two independent [CuI(PPh_3_)_2_(ptu)] molecules in the asymmetric unit. The molecules feature a distorted tetrahedral copper(I) center with two triphenylphosphine P atoms, one S atom from ptu ligand and one iodide atom (Figures 1, 2). A distorted tetrahedral geometry is also found in the copper(I) halide complexes with mixed PPh_3_/sulfur base ligands (Lecomte *et al.*, 1989; Karagiannidis *et al.*, 1990; Skoulika *et al.*, 1991; Jianping & Kazuyuki., 1996; Aslanidis *et al.*, 1998; Cox *et al.*, 1999). The P—Cu—P angle deviates considerably from tetrahedral value at 118.63 (5)° and 122.18 (5)° in molecule A and B, respectively. The Cu—P distances of the molecule A [Cu(1)—P(1), 2.2908 (15)Å and Cu(1)—P(2), 2.3024 (16) Å] and of the molecule B [Cu(2)—P(3), 2.2876 (15)Å and Cu(2)—P(4), 2.2974 (16) Å] are slightly shorter as compared to the observed value for [Cu(PPh_3_)_3_I] (Bowmaker *et al.*, 1987). The observed Cu—S distances of 2.4148 (16) and 2.3942 (15) Å in molecule A and B are consistent with the distances usually found for tetrahedrally coordinated copper(I) with thioamide-sulfur donors (Aslanidis *et al.*, 1993). The mean plane of the phenyl ring (C45—C50) of ptu ligand in the molecule B forms a dihedral angle of 83.9 (2)° with one of phenyl ring (C81—C86) of PPh_3_ molecules. Weak intra-molecular hydrogen bonding between the amide group and iodide atom is observed [N2···I1= 3.511 (5) Å, H2A···I1 = 2.65 Å, N2—H2A···I1 = 166 ° for molecule A and N4···I2 = 3.567 (4) Å, H4A···I2 = 2.71 Å, N4—H4A···I2 = 165 ° for molecule B]. However, only one C—H···π interaction between C-*sp*^2^ (C46—H46···π) of the phenyl ring from ptu ligand and the centroid of one phenyl ring (*Cg*14, C81—C86) of the PPh_3_ molecules is observed in molecule B. In addition, the weak inter-molecular interactions between the one H atom of NH_2_ group of ptu ligand of molecule A and the thione-S atom of ptu of molecule B [N1···S2 = 3.455 (5) Å, H1B···S2 = 2.58 Å, N1—H1B···S2 = 173 °] are found and *vice versa* [N3···S1 = 3.396 (5) Å, H3B···S1 = 2.63 Å, N3—H3B···S1 = 146 °]. The intra- and inter-molecular interactions of this complex are shown in Figure 3.

### Experimental

Triphenylphosphine (0.27 g, 1.05 mmol) was dissolved in 30 cm^3 ^of acetonitrile at 70–75°C. CuI (0.1 g, 0.52 mmol) was added and the mixture was stirred for 2 h. After the formation of a complete clear solution, *N*-phenylthiourea (0.24 g, 1.57 mmol) was added slowly and the reaction mixture was stirring for 3 h. The resulting clear solution was filtered off and left to evaporate at room temperature. The microcrystalline solid, which deposited upon standing for several days, was filtered off and dried *in vacuo*. Single crystals suitable for X-ray diffraction studies were obtained by slow evaporation in acetonitrile. The melting point of the complex is 456-457 K. Elemental analysis, calculated for [CuI(PPh_3_)_2_(ptu)]: C, 59.55; H, 4.43; N, 3.23; S, 3.70%, found: C, 60.28; H, 4.46; N, 3.56; S, 3.93%.

### Refinement

All H atoms atoms were constrained with a riding model for C-*sp*^2^ [C—H = 0.95 Å and with *U*_iso_(H) = 1.2*U*_eq_(C)] and for N atoms [N—H = 0.88 Å and with *U*_iso_(H) = 1.2*U*_eq_(N).

### Crystal data

**Table:**

[CuI(C_18_H_15_P)_2_(C_7_H_8_N_2_S)]	*Z* = 4
*M**_r_* = 867.19	*F*_000_ = 1752
Triclinic, *P*1	*D*_x_ = 1.498 Mg m^−^^3^
Hall symbol: -P 1	Mo *K*α radiation λ = 0.71074 Å
*a* = 10.9505 (9) Å	Cell parameters from 8397 reflections
*b* = 18.7294 (15) Å	θ = 2.4–27.1º
*c* = 21.3731 (18) Å	µ = 1.54 mm^−^^1^
α = 67.422 (1)º	*T* = 173 (2) K
β = 77.215 (1)º	Plate, colorless
γ = 73.224 (1)º	0.50 × 0.30 × 0.07 mm
*V* = 3844.9 (5) Å^3^	

### Data collection

**Table:**

Bruker Platform diffractometer	15698 independent reflections
Monochromator: graphite	8887 reflections with *I* > 2s(*I*)
Detector resolution: 8.192 pixels mm^-1^	*R*_int_ = 0.089
*T* = 173 K	θ_max_ = 26.4º
ω scans	θ_min_ = 1.0º
Absorption correction: multi-scan(SADABS; Bruker, 2003)	*h* = −13→13
*T*_min_ = 0.582, *T*_max_ = 0.898	*k* = −23→23
42789 measured reflections	*l* = −26→26

### Refinement

**Table:**

Refinement on *F*^2^	Secondary atom site location: difference Fourier map
Least-squares matrix: full	Hydrogen site location: inferred from neighbouring sites
*R*[*F*^2^ > 2σ(*F*^2^)] = 0.050	H-atom parameters constrained
*wR*(*F*^2^) = 0.108	*w* = 1/[σ^2^(*F*_o_^2^) + (0.0284*P*)^2^ + 1.9087*P*] where *P* = (*F*_o_^2^ + 2*F*_c_^2^)/3
*S* = 0.98	(Δ/σ)_max_ = 0.001
15698 reflections	Δρ_max_ = 0.82 e Å^−^^3^
901 parameters	Δρ_min_ = −0.74 e Å^−^^3^
Primary atom site location: structure-invariant direct methods	Extinction correction: none

### Special details

**Table:**

Geometry. All e.s.d.'s (except the e.s.d. in the dihedral angle between two l.s. planes) are estimated using the full covariance matrix. The cell e.s.d.'s are taken into account individually in the estimation of e.s.d.'s in distances, angles and torsion angles; correlations between e.s.d.'s in cell parameters are only used when they are defined by crystal symmetry. An approximate (isotropic) treatment of cell e.s.d.'s is used for estimating e.s.d.'s involving l.s. planes.
Refinement. Refinement of *F*^2^ against ALL reflections. The weighted *R*-factor *wR* and goodness of fit *S* are based on *F*^2^, conventional *R*-factors *R* are based on *F*, with *F* set to zero for negative *F*^2^. The threshold expression of *F*^2^ > σ(*F*^2^) is used only for calculating *R*-factors(gt) *etc*. and is not relevant to the choice of reflections for refinement. *R*-factors based on *F*^2^ are statistically about twice as large as those based on *F*, and *R*- factors based on ALL data will be even larger.

### Fractional atomic coordinates and isotropic or equivalent isotropic displacement parameters (Å^2^)

**Table:**

	*x*	*y*	*z*	*U*_iso_*/*U*_eq_	
Cu1	0.22484 (6)	0.77723 (4)	0.37656 (3)	0.02320 (17)	
Cu2	0.80632 (6)	0.24103 (4)	0.10985 (3)	0.02128 (16)	
I1	0.46394 (3)	0.75683 (2)	0.39996 (2)	0.02967 (11)	
I2	0.56365 (3)	0.26189 (2)	0.09357 (2)	0.02911 (11)	
S1	0.15450 (14)	0.90317 (8)	0.28957 (8)	0.0300 (4)	
S2	0.86425 (13)	0.12053 (8)	0.20281 (7)	0.0228 (3)	
N1	0.1515 (4)	1.0517 (3)	0.2712 (2)	0.0332 (12)	
H1A	0.1840	1.0886	0.2729	0.040*	
H1B	0.0778	1.0648	0.2550	0.040*	
N2	0.3254 (4)	0.9573 (3)	0.3173 (2)	0.0316 (12)	
H2A	0.3623	0.9064	0.3303	0.038*	
N3	0.8570 (4)	−0.0299 (2)	0.2387 (2)	0.0316 (12)	
H3A	0.8305	−0.0692	0.2371	0.038*	
H3B	0.9134	−0.0396	0.2662	0.038*	
N4	0.7271 (4)	0.0587 (2)	0.1580 (2)	0.0267 (11)	
H4A	0.6980	0.1089	0.1346	0.032*	
P1	0.21516 (14)	0.68413 (8)	0.33399 (7)	0.0217 (3)	
P2	0.11408 (14)	0.77457 (8)	0.48212 (7)	0.0229 (3)	
P3	0.80062 (13)	0.33467 (8)	0.15518 (7)	0.0212 (3)	
P4	0.93858 (14)	0.23217 (8)	0.01210 (7)	0.0214 (3)	
C1	0.2138 (5)	0.9760 (3)	0.2929 (3)	0.0246 (13)	
C2	0.3955 (5)	1.0065 (3)	0.3258 (3)	0.0297 (14)	
C3	0.4523 (5)	0.9764 (3)	0.3850 (3)	0.0332 (15)	
H3	0.4354	0.9292	0.4201	0.040*	
C4	0.5344 (5)	1.0157 (3)	0.3924 (3)	0.0376 (16)	
H4	0.5749	0.9948	0.4327	0.045*	
C5	0.5582 (6)	1.0846 (3)	0.3425 (3)	0.0375 (16)	
H5	0.6150	1.1111	0.3480	0.045*	
C6	0.4991 (6)	1.1147 (3)	0.2848 (3)	0.0366 (16)	
H6	0.5137	1.1632	0.2510	0.044*	
C7	0.4180 (5)	1.0764 (3)	0.2742 (3)	0.0326 (15)	
H7	0.3793	1.0971	0.2333	0.039*	
C8	0.3242 (5)	0.6753 (3)	0.2570 (3)	0.0247 (13)	
C9	0.4422 (6)	0.6943 (4)	0.2459 (3)	0.0379 (16)	
H9	0.4596	0.7179	0.2742	0.045*	
C10	0.5348 (6)	0.6796 (4)	0.1941 (3)	0.0454 (18)	
H10	0.6159	0.6919	0.1877	0.054*	
C11	0.5100 (6)	0.6469 (3)	0.1513 (3)	0.0417 (17)	
H11	0.5736	0.6364	0.1157	0.050*	
C12	0.3918 (6)	0.6299 (3)	0.1613 (3)	0.0363 (15)	
H12	0.3736	0.6082	0.1318	0.044*	
C13	0.2983 (5)	0.6438 (3)	0.2136 (3)	0.0284 (14)	
H13	0.2171	0.6318	0.2196	0.034*	
C14	0.0541 (5)	0.7024 (3)	0.3118 (3)	0.0222 (13)	
C15	0.0182 (5)	0.7605 (3)	0.2507 (3)	0.0262 (13)	
H15	0.0805	0.7864	0.2184	0.031*	
C16	−0.1063 (5)	0.7808 (3)	0.2361 (3)	0.0268 (13)	
H16	−0.1293	0.8209	0.1943	0.032*	
C17	−0.1984 (5)	0.7431 (3)	0.2824 (3)	0.0317 (15)	
H17	−0.2841	0.7569	0.2720	0.038*	
C18	−0.1649 (5)	0.6852 (3)	0.3436 (3)	0.0301 (14)	
H18	−0.2275	0.6593	0.3755	0.036*	
C19	−0.0399 (5)	0.6652 (3)	0.3582 (3)	0.0239 (13)	
H19	−0.0175	0.6257	0.4004	0.029*	
C20	0.2465 (5)	0.5804 (3)	0.3896 (3)	0.0234 (13)	
C21	0.2035 (5)	0.5231 (3)	0.3786 (3)	0.0267 (13)	
H21	0.1548	0.5381	0.3421	0.032*	
C22	0.2318 (5)	0.4449 (3)	0.4208 (3)	0.0314 (14)	
H22	0.2000	0.4068	0.4138	0.038*	
C23	0.3049 (6)	0.4212 (3)	0.4727 (3)	0.0387 (16)	
H23	0.3255	0.3668	0.5006	0.046*	
C24	0.3487 (6)	0.4768 (3)	0.4842 (3)	0.0395 (16)	
H24	0.3989	0.4609	0.5203	0.047*	
C25	0.3192 (5)	0.5558 (3)	0.4431 (3)	0.0309 (14)	
H25	0.3491	0.5938	0.4515	0.037*	
C26	−0.0620 (5)	0.7959 (3)	0.4911 (3)	0.0250 (13)	
C27	−0.1209 (5)	0.8044 (3)	0.4374 (3)	0.0298 (14)	
H27	−0.0702	0.8021	0.3957	0.036*	
C28	−0.2544 (6)	0.8164 (4)	0.4434 (3)	0.0406 (16)	
H28	−0.2947	0.8232	0.4058	0.049*	
C29	−0.3271 (6)	0.8183 (3)	0.5047 (3)	0.0399 (16)	
H29	−0.4178	0.8253	0.5097	0.048*	
C30	−0.2687 (6)	0.8102 (3)	0.5582 (3)	0.0383 (16)	
H30	−0.3193	0.8130	0.5998	0.046*	
C31	−0.1364 (5)	0.7979 (3)	0.5520 (3)	0.0333 (15)	
H31	−0.0965	0.7907	0.5899	0.040*	
C32	0.1544 (5)	0.6755 (3)	0.5464 (3)	0.0253 (13)	
C33	0.0754 (6)	0.6219 (3)	0.5641 (3)	0.0368 (16)	
H33	−0.0039	0.6385	0.5460	0.044*	
C34	0.1128 (7)	0.5448 (4)	0.6079 (3)	0.0501 (19)	
H34	0.0594	0.5086	0.6190	0.060*	
C35	0.2258 (7)	0.5201 (4)	0.6355 (3)	0.0477 (18)	
H35	0.2491	0.4674	0.6664	0.057*	
C36	0.3056 (7)	0.5711 (4)	0.6186 (3)	0.0454 (17)	
H36	0.3845	0.5537	0.6373	0.054*	
C37	0.2700 (6)	0.6486 (3)	0.5740 (3)	0.0333 (15)	
H37	0.3257	0.6836	0.5623	0.040*	
C38	0.1383 (5)	0.8424 (3)	0.5191 (3)	0.0277 (14)	
C39	0.1311 (6)	0.8262 (4)	0.5887 (3)	0.0401 (16)	
H39	0.1221	0.7755	0.6205	0.048*	
C40	0.1373 (7)	0.8861 (4)	0.6111 (4)	0.055 (2)	
H40	0.1299	0.8760	0.6587	0.066*	
C41	0.1542 (6)	0.9597 (4)	0.5653 (4)	0.054 (2)	
H41	0.1597	0.9994	0.5813	0.065*	
C42	0.1626 (7)	0.9743 (4)	0.4973 (4)	0.056 (2)	
H42	0.1750	1.0243	0.4655	0.067*	
C43	0.1534 (6)	0.9167 (4)	0.4743 (3)	0.0407 (17)	
H43	0.1575	0.9283	0.4267	0.049*	
C44	0.8119 (5)	0.0442 (3)	0.1994 (3)	0.0208 (12)	
C45	0.6767 (5)	0.0025 (3)	0.1467 (3)	0.0239 (13)	
C46	0.6858 (5)	0.0049 (3)	0.0809 (3)	0.0305 (14)	
H46	0.7283	0.0415	0.0446	0.037*	
C47	0.6331 (6)	−0.0459 (4)	0.0672 (3)	0.0382 (16)	
H47	0.6404	−0.0445	0.0216	0.046*	
C48	0.5700 (6)	−0.0984 (4)	0.1198 (3)	0.0386 (16)	
H48	0.5331	−0.1328	0.1105	0.046*	
C49	0.5607 (5)	−0.1007 (3)	0.1860 (3)	0.0388 (16)	
H49	0.5177	−0.1370	0.2223	0.047*	
C50	0.6145 (5)	−0.0498 (3)	0.2000 (3)	0.0263 (13)	
H50	0.6082	−0.0514	0.2455	0.032*	
C51	0.6712 (5)	0.3423 (3)	0.2242 (3)	0.0246 (13)	
C52	0.6550 (6)	0.2738 (3)	0.2773 (3)	0.0447 (18)	
H52	0.7103	0.2245	0.2765	0.054*	
C53	0.5600 (7)	0.2748 (4)	0.3319 (3)	0.053 (2)	
H53	0.5545	0.2271	0.3693	0.064*	
C54	0.4746 (6)	0.3436 (4)	0.3325 (4)	0.0490 (19)	
H54	0.4053	0.3436	0.3682	0.059*	
C55	0.4896 (6)	0.4126 (4)	0.2810 (4)	0.056 (2)	
H55	0.4321	0.4612	0.2818	0.067*	
C56	0.5880 (6)	0.4124 (4)	0.2276 (3)	0.0464 (18)	
H56	0.5984	0.4612	0.1928	0.056*	
C57	0.9462 (5)	0.3192 (3)	0.1916 (3)	0.0199 (12)	
C58	1.0621 (5)	0.2804 (3)	0.1645 (3)	0.0268 (13)	
H58	1.0629	0.2610	0.1293	0.032*	
C59	1.1759 (6)	0.2696 (3)	0.1883 (3)	0.0349 (15)	
H59	1.2547	0.2453	0.1678	0.042*	
C60	1.1761 (6)	0.2939 (3)	0.2413 (3)	0.0340 (15)	
H60	1.2544	0.2854	0.2581	0.041*	
C61	1.0622 (6)	0.3306 (3)	0.2698 (3)	0.0326 (15)	
H61	1.0621	0.3466	0.3070	0.039*	
C62	0.9478 (6)	0.3446 (3)	0.2453 (3)	0.0271 (13)	
H62	0.8703	0.3714	0.2647	0.033*	
C63	0.7774 (5)	0.4363 (3)	0.0956 (3)	0.0225 (13)	
C64	0.8678 (5)	0.4821 (3)	0.0775 (3)	0.0284 (14)	
H64	0.9438	0.4619	0.0990	0.034*	
C65	0.8473 (6)	0.5571 (3)	0.0279 (3)	0.0356 (15)	
H65	0.9102	0.5877	0.0150	0.043*	
C66	0.7355 (6)	0.5873 (3)	−0.0025 (3)	0.0353 (15)	
H66	0.7216	0.6388	−0.0362	0.042*	
C67	0.6440 (6)	0.5429 (3)	0.0158 (3)	0.0328 (15)	
H67	0.5667	0.5640	−0.0046	0.039*	
C68	0.6657 (6)	0.4678 (3)	0.0640 (3)	0.0289 (14)	
H68	0.6034	0.4370	0.0758	0.035*	
C69	1.1067 (5)	0.1898 (3)	0.0278 (3)	0.0204 (12)	
C70	1.2036 (5)	0.2323 (3)	−0.0020 (3)	0.0297 (14)	
H70	1.1860	0.2828	−0.0368	0.036*	
C71	1.3245 (6)	0.2011 (4)	0.0189 (3)	0.0370 (16)	
H71	1.3895	0.2304	−0.0019	0.044*	
C72	1.3524 (6)	0.1279 (4)	0.0696 (3)	0.0351 (15)	
H72	1.4354	0.1074	0.0841	0.042*	
C73	1.2581 (5)	0.0846 (3)	0.0989 (3)	0.0307 (14)	
H73	1.2767	0.0338	0.1331	0.037*	
C74	1.1364 (5)	0.1156 (3)	0.0781 (3)	0.0273 (13)	
H74	1.0723	0.0857	0.0985	0.033*	
C75	0.9415 (5)	0.3227 (3)	−0.0624 (3)	0.0242 (13)	
C76	0.8565 (6)	0.3940 (3)	−0.0611 (3)	0.0316 (14)	
H76	0.8008	0.3963	−0.0206	0.038*	
C77	0.8536 (7)	0.4618 (4)	−0.1195 (3)	0.0461 (18)	
H77	0.7960	0.5103	−0.1184	0.055*	
C78	0.9325 (7)	0.4593 (4)	−0.1779 (3)	0.0484 (19)	
H78	0.9295	0.5060	−0.2172	0.058*	
C79	1.0163 (7)	0.3897 (4)	−0.1803 (3)	0.0461 (18)	
H79	1.0716	0.3884	−0.2211	0.055*	
C80	1.0202 (6)	0.3215 (3)	−0.1234 (3)	0.0366 (15)	
H80	1.0771	0.2733	−0.1257	0.044*	
C81	0.9045 (5)	0.1720 (3)	−0.0295 (3)	0.0194 (12)	
C82	0.9928 (5)	0.1096 (3)	−0.0451 (3)	0.0236 (13)	
H82	1.0759	0.0925	−0.0308	0.028*	
C83	0.9595 (6)	0.0719 (3)	−0.0819 (3)	0.0313 (14)	
H83	1.0204	0.0291	−0.0925	0.038*	
C84	0.8404 (6)	0.0960 (3)	−0.1029 (3)	0.0312 (15)	
H84	0.8191	0.0699	−0.1280	0.037*	
C85	0.7513 (6)	0.1577 (3)	−0.0879 (3)	0.0305 (14)	
H85	0.6688	0.1746	−0.1028	0.037*	
C86	0.7827 (5)	0.1956 (3)	−0.0506 (3)	0.0287 (14)	
H86	0.7208	0.2377	−0.0394	0.034*	

### Atomic displacement parameters (Å^2^)

**Table:**

	*U*^11^	*U*^22^	*U*^33^	*U*^12^	*U*^13^	*U*^23^
Cu1	0.0238 (4)	0.0223 (4)	0.0256 (4)	−0.0074 (3)	−0.0024 (3)	−0.0092 (3)
Cu2	0.0239 (4)	0.0165 (3)	0.0254 (4)	−0.0047 (3)	−0.0050 (3)	−0.0082 (3)
I1	0.0237 (2)	0.0261 (2)	0.0380 (3)	−0.00612 (18)	−0.00825 (18)	−0.00693 (19)
I2	0.0247 (2)	0.0208 (2)	0.0409 (3)	−0.00426 (17)	−0.01189 (18)	−0.00596 (18)
S1	0.0375 (9)	0.0223 (8)	0.0324 (9)	−0.0072 (7)	−0.0164 (7)	−0.0048 (7)
S2	0.0289 (8)	0.0168 (7)	0.0249 (8)	−0.0047 (6)	−0.0088 (6)	−0.0070 (6)
N1	0.035 (3)	0.025 (3)	0.044 (3)	−0.006 (2)	−0.017 (2)	−0.010 (2)
N2	0.035 (3)	0.020 (3)	0.044 (3)	−0.010 (2)	−0.012 (3)	−0.008 (2)
N3	0.044 (3)	0.014 (2)	0.040 (3)	−0.005 (2)	−0.021 (3)	−0.004 (2)
N4	0.030 (3)	0.014 (2)	0.036 (3)	−0.001 (2)	−0.017 (2)	−0.005 (2)
P1	0.0254 (8)	0.0174 (7)	0.0224 (8)	−0.0066 (7)	−0.0023 (7)	−0.0061 (6)
P2	0.0250 (8)	0.0230 (8)	0.0232 (8)	−0.0082 (7)	−0.0017 (7)	−0.0095 (7)
P3	0.0237 (8)	0.0158 (7)	0.0251 (8)	−0.0041 (6)	−0.0041 (7)	−0.0078 (6)
P4	0.0250 (8)	0.0178 (8)	0.0236 (8)	−0.0061 (6)	−0.0043 (7)	−0.0077 (7)
C1	0.034 (4)	0.019 (3)	0.019 (3)	−0.003 (3)	−0.005 (3)	−0.005 (3)
C2	0.028 (3)	0.028 (3)	0.034 (4)	−0.004 (3)	−0.005 (3)	−0.012 (3)
C3	0.037 (4)	0.028 (3)	0.036 (4)	−0.014 (3)	−0.009 (3)	−0.005 (3)
C4	0.031 (4)	0.035 (4)	0.050 (4)	−0.011 (3)	−0.008 (3)	−0.015 (3)
C5	0.040 (4)	0.028 (4)	0.051 (4)	−0.016 (3)	−0.006 (3)	−0.015 (3)
C6	0.038 (4)	0.023 (3)	0.045 (4)	−0.013 (3)	0.004 (3)	−0.009 (3)
C7	0.033 (4)	0.028 (3)	0.033 (4)	−0.009 (3)	−0.003 (3)	−0.005 (3)
C8	0.027 (3)	0.016 (3)	0.028 (3)	−0.006 (3)	0.002 (3)	−0.004 (3)
C9	0.042 (4)	0.045 (4)	0.030 (4)	−0.019 (3)	0.009 (3)	−0.018 (3)
C10	0.037 (4)	0.061 (5)	0.047 (4)	−0.024 (4)	0.013 (3)	−0.030 (4)
C11	0.057 (5)	0.029 (4)	0.032 (4)	−0.006 (3)	0.012 (3)	−0.013 (3)
C12	0.052 (4)	0.025 (3)	0.032 (4)	−0.005 (3)	−0.004 (3)	−0.014 (3)
C13	0.030 (3)	0.023 (3)	0.032 (4)	−0.006 (3)	0.000 (3)	−0.011 (3)
C14	0.025 (3)	0.020 (3)	0.026 (3)	−0.002 (3)	0.000 (3)	−0.016 (3)
C15	0.033 (3)	0.023 (3)	0.027 (3)	−0.011 (3)	−0.001 (3)	−0.011 (3)
C16	0.031 (3)	0.022 (3)	0.027 (3)	−0.002 (3)	−0.009 (3)	−0.009 (3)
C17	0.023 (3)	0.036 (4)	0.045 (4)	−0.001 (3)	−0.012 (3)	−0.023 (3)
C18	0.030 (3)	0.030 (3)	0.033 (4)	−0.014 (3)	0.003 (3)	−0.012 (3)
C19	0.037 (4)	0.013 (3)	0.019 (3)	−0.004 (3)	−0.003 (3)	−0.003 (2)
C20	0.020 (3)	0.017 (3)	0.027 (3)	−0.002 (2)	0.003 (3)	−0.006 (3)
C21	0.024 (3)	0.019 (3)	0.033 (4)	−0.003 (3)	−0.001 (3)	−0.009 (3)
C22	0.028 (3)	0.021 (3)	0.043 (4)	−0.010 (3)	0.005 (3)	−0.011 (3)
C23	0.043 (4)	0.016 (3)	0.044 (4)	−0.003 (3)	0.007 (3)	−0.006 (3)
C24	0.048 (4)	0.029 (4)	0.029 (4)	0.004 (3)	−0.010 (3)	−0.002 (3)
C25	0.037 (4)	0.026 (3)	0.028 (3)	−0.001 (3)	−0.003 (3)	−0.011 (3)
C26	0.021 (3)	0.022 (3)	0.035 (4)	−0.006 (3)	−0.004 (3)	−0.012 (3)
C27	0.026 (3)	0.033 (3)	0.036 (4)	−0.007 (3)	0.002 (3)	−0.019 (3)
C28	0.039 (4)	0.045 (4)	0.044 (4)	−0.006 (3)	−0.019 (3)	−0.016 (3)
C29	0.025 (4)	0.037 (4)	0.052 (5)	−0.008 (3)	−0.001 (3)	−0.012 (3)
C30	0.036 (4)	0.041 (4)	0.030 (4)	−0.007 (3)	0.001 (3)	−0.008 (3)
C31	0.030 (4)	0.044 (4)	0.029 (4)	−0.008 (3)	−0.008 (3)	−0.014 (3)
C32	0.026 (3)	0.021 (3)	0.027 (3)	−0.005 (3)	0.005 (3)	−0.011 (3)
C33	0.034 (4)	0.026 (3)	0.049 (4)	−0.004 (3)	0.000 (3)	−0.016 (3)
C34	0.059 (5)	0.027 (4)	0.058 (5)	−0.018 (4)	0.008 (4)	−0.010 (4)
C35	0.057 (5)	0.029 (4)	0.042 (4)	−0.007 (4)	−0.003 (4)	0.001 (3)
C36	0.053 (4)	0.038 (4)	0.039 (4)	−0.005 (4)	−0.016 (3)	−0.004 (3)
C37	0.036 (4)	0.037 (4)	0.031 (4)	−0.008 (3)	−0.007 (3)	−0.014 (3)
C38	0.027 (3)	0.030 (3)	0.030 (4)	−0.009 (3)	0.001 (3)	−0.014 (3)
C39	0.054 (4)	0.037 (4)	0.033 (4)	−0.008 (3)	−0.014 (3)	−0.012 (3)
C40	0.066 (5)	0.065 (5)	0.051 (5)	−0.007 (4)	−0.024 (4)	−0.035 (4)
C41	0.044 (4)	0.063 (5)	0.086 (6)	−0.008 (4)	−0.012 (4)	−0.060 (5)
C42	0.070 (5)	0.046 (5)	0.071 (6)	−0.027 (4)	0.007 (4)	−0.038 (4)
C43	0.055 (4)	0.038 (4)	0.037 (4)	−0.021 (3)	0.009 (3)	−0.022 (3)
C44	0.018 (3)	0.017 (3)	0.023 (3)	−0.003 (2)	−0.002 (2)	−0.004 (3)
C45	0.026 (3)	0.017 (3)	0.029 (3)	−0.006 (3)	−0.006 (3)	−0.006 (3)
C46	0.031 (3)	0.029 (3)	0.029 (4)	−0.006 (3)	−0.003 (3)	−0.009 (3)
C47	0.040 (4)	0.045 (4)	0.044 (4)	−0.010 (3)	−0.006 (3)	−0.030 (3)
C48	0.034 (4)	0.038 (4)	0.058 (5)	−0.013 (3)	−0.011 (3)	−0.025 (4)
C49	0.029 (4)	0.029 (4)	0.057 (5)	−0.015 (3)	−0.001 (3)	−0.010 (3)
C50	0.027 (3)	0.026 (3)	0.024 (3)	−0.006 (3)	−0.003 (3)	−0.008 (3)
C51	0.031 (3)	0.018 (3)	0.028 (3)	−0.007 (3)	−0.006 (3)	−0.010 (3)
C52	0.052 (4)	0.023 (4)	0.051 (4)	−0.005 (3)	0.016 (4)	−0.019 (3)
C53	0.070 (5)	0.033 (4)	0.049 (5)	−0.023 (4)	0.028 (4)	−0.017 (4)
C54	0.041 (4)	0.046 (5)	0.057 (5)	−0.010 (4)	0.018 (4)	−0.029 (4)
C55	0.050 (5)	0.041 (4)	0.057 (5)	0.015 (4)	0.009 (4)	−0.023 (4)
C56	0.058 (5)	0.025 (4)	0.035 (4)	0.008 (3)	0.000 (3)	−0.003 (3)
C57	0.027 (3)	0.013 (3)	0.019 (3)	−0.006 (2)	−0.004 (2)	−0.003 (2)
C58	0.032 (3)	0.018 (3)	0.031 (3)	−0.004 (3)	−0.003 (3)	−0.012 (3)
C59	0.024 (3)	0.039 (4)	0.039 (4)	−0.003 (3)	−0.005 (3)	−0.012 (3)
C60	0.040 (4)	0.026 (3)	0.035 (4)	−0.009 (3)	−0.020 (3)	−0.001 (3)
C61	0.047 (4)	0.025 (3)	0.025 (3)	−0.008 (3)	−0.012 (3)	−0.004 (3)
C62	0.038 (4)	0.023 (3)	0.021 (3)	−0.010 (3)	−0.002 (3)	−0.005 (3)
C63	0.032 (3)	0.018 (3)	0.023 (3)	−0.006 (3)	−0.006 (3)	−0.010 (3)
C64	0.033 (3)	0.026 (3)	0.027 (3)	−0.007 (3)	−0.008 (3)	−0.007 (3)
C65	0.033 (4)	0.024 (3)	0.048 (4)	−0.012 (3)	0.001 (3)	−0.009 (3)
C66	0.047 (4)	0.017 (3)	0.034 (4)	−0.002 (3)	−0.004 (3)	−0.005 (3)
C67	0.034 (4)	0.023 (3)	0.039 (4)	0.001 (3)	−0.017 (3)	−0.007 (3)
C68	0.041 (4)	0.019 (3)	0.032 (4)	−0.010 (3)	−0.009 (3)	−0.009 (3)
C69	0.023 (3)	0.024 (3)	0.020 (3)	−0.008 (3)	−0.002 (2)	−0.013 (3)
C70	0.039 (4)	0.027 (3)	0.032 (4)	−0.012 (3)	−0.004 (3)	−0.015 (3)
C71	0.034 (4)	0.033 (4)	0.054 (4)	−0.015 (3)	−0.006 (3)	−0.019 (3)
C72	0.027 (3)	0.044 (4)	0.045 (4)	0.003 (3)	−0.014 (3)	−0.029 (3)
C73	0.033 (4)	0.029 (3)	0.030 (4)	0.006 (3)	−0.010 (3)	−0.015 (3)
C74	0.035 (4)	0.030 (3)	0.024 (3)	−0.011 (3)	−0.002 (3)	−0.015 (3)
C75	0.033 (3)	0.022 (3)	0.020 (3)	−0.009 (3)	−0.007 (3)	−0.006 (3)
C76	0.043 (4)	0.023 (3)	0.030 (4)	0.000 (3)	−0.010 (3)	−0.013 (3)
C77	0.073 (5)	0.022 (4)	0.041 (4)	−0.006 (3)	−0.012 (4)	−0.009 (3)
C78	0.083 (6)	0.025 (4)	0.039 (4)	−0.027 (4)	−0.014 (4)	0.002 (3)
C79	0.072 (5)	0.040 (4)	0.027 (4)	−0.028 (4)	0.004 (3)	−0.007 (3)
C80	0.054 (4)	0.026 (3)	0.030 (4)	−0.009 (3)	−0.010 (3)	−0.008 (3)
C81	0.023 (3)	0.016 (3)	0.016 (3)	−0.006 (2)	−0.003 (2)	−0.001 (2)
C82	0.032 (3)	0.017 (3)	0.025 (3)	−0.011 (3)	−0.006 (3)	−0.005 (3)
C83	0.048 (4)	0.020 (3)	0.028 (3)	−0.011 (3)	0.000 (3)	−0.009 (3)
C84	0.059 (4)	0.018 (3)	0.025 (3)	−0.019 (3)	−0.010 (3)	−0.005 (3)
C85	0.039 (4)	0.030 (3)	0.026 (3)	−0.018 (3)	−0.015 (3)	0.000 (3)
C86	0.041 (4)	0.019 (3)	0.025 (3)	−0.011 (3)	−0.004 (3)	−0.004 (3)

### Geometric parameters (Å, °)

**Table:**

Cu1—P1	2.2908 (15)	C34—H34	0.9500
Cu1—P2	2.3024 (16)	C35—C36	1.373 (8)
Cu1—S1	2.4148 (16)	C35—H35	0.9500
Cu1—I1	2.6658 (8)	C36—C37	1.393 (8)
Cu2—P3	2.2876 (15)	C36—H36	0.9500
Cu2—P4	2.2974 (16)	C37—H37	0.9500
Cu2—S2	2.3942 (15)	C38—C43	1.385 (8)
Cu2—I2	2.6534 (8)	C38—C39	1.387 (7)
S1—C1	1.701 (5)	C39—C40	1.399 (8)
S2—C44	1.718 (5)	C39—H39	0.9500
N1—C1	1.329 (6)	C40—C41	1.388 (9)
N1—H1A	0.8800	C40—H40	0.9500
N1—H1B	0.8800	C41—C42	1.358 (9)
N2—C1	1.335 (6)	C41—H41	0.9500
N2—C2	1.438 (7)	C42—C43	1.381 (8)
N2—H2A	0.8800	C42—H42	0.9500
N3—C44	1.328 (6)	C43—H43	0.9500
N3—H3A	0.8800	C45—C46	1.371 (7)
N3—H3B	0.8800	C45—C50	1.382 (7)
N4—C44	1.324 (6)	C46—C47	1.391 (7)
N4—C45	1.434 (6)	C46—H46	0.9500
N4—H4A	0.8800	C47—C48	1.381 (8)
P1—C20	1.824 (5)	C47—H47	0.9500
P1—C14	1.830 (5)	C48—C49	1.380 (8)
P1—C8	1.841 (5)	C48—H48	0.9500
P2—C32	1.833 (5)	C49—C50	1.402 (7)
P2—C26	1.834 (5)	C49—H49	0.9500
P2—C38	1.836 (5)	C50—H50	0.9500
P3—C63	1.821 (5)	C51—C52	1.376 (7)
P3—C51	1.824 (6)	C51—C56	1.383 (7)
P3—C57	1.827 (5)	C52—C53	1.382 (8)
P4—C75	1.830 (5)	C52—H52	0.9500
P4—C69	1.833 (5)	C53—C54	1.360 (8)
P4—C81	1.834 (5)	C53—H53	0.9500
C2—C3	1.379 (7)	C54—C55	1.363 (9)
C2—C7	1.396 (7)	C54—H54	0.9500
C3—C4	1.383 (7)	C55—C56	1.386 (8)
C3—H3	0.9500	C55—H55	0.9500
C4—C5	1.373 (8)	C56—H56	0.9500
C4—H4	0.9500	C57—C58	1.393 (7)
C5—C6	1.366 (8)	C57—C62	1.407 (7)
C5—H5	0.9500	C58—C59	1.382 (7)
C6—C7	1.391 (7)	C58—H58	0.9500
C6—H6	0.9500	C59—C60	1.374 (7)
C7—H7	0.9500	C59—H59	0.9500
C8—C9	1.385 (7)	C60—C61	1.376 (8)
C8—C13	1.386 (7)	C60—H60	0.9500
C9—C10	1.381 (8)	C61—C62	1.381 (7)
C9—H9	0.9500	C61—H61	0.9500
C10—C11	1.385 (8)	C62—H62	0.9500
C10—H10	0.9500	C63—C64	1.388 (7)
C11—C12	1.373 (8)	C63—C68	1.395 (7)
C11—H11	0.9500	C64—C65	1.387 (7)
C12—C13	1.388 (7)	C64—H64	0.9500
C12—H12	0.9500	C65—C66	1.383 (8)
C13—H13	0.9500	C65—H65	0.9500
C14—C15	1.391 (7)	C66—C67	1.380 (7)
C14—C19	1.399 (7)	C66—H66	0.9500
C15—C16	1.377 (7)	C67—C68	1.377 (7)
C15—H15	0.9500	C67—H67	0.9500
C16—C17	1.389 (7)	C68—H68	0.9500
C16—H16	0.9500	C69—C74	1.392 (7)
C17—C18	1.384 (8)	C69—C70	1.401 (7)
C17—H17	0.9500	C70—C71	1.381 (8)
C18—C19	1.384 (7)	C70—H70	0.9500
C18—H18	0.9500	C71—C72	1.383 (8)
C19—H19	0.9500	C71—H71	0.9500
C20—C25	1.392 (7)	C72—C73	1.387 (8)
C20—C21	1.402 (7)	C72—H72	0.9500
C21—C22	1.379 (7)	C73—C74	1.388 (7)
C21—H21	0.9500	C73—H73	0.9500
C22—C23	1.370 (8)	C74—H74	0.9500
C22—H22	0.9500	C75—C76	1.396 (7)
C23—C24	1.384 (8)	C75—C80	1.398 (7)
C23—H23	0.9500	C76—C77	1.396 (8)
C24—C25	1.385 (7)	C76—H76	0.9500
C24—H24	0.9500	C77—C78	1.361 (8)
C25—H25	0.9500	C77—H77	0.9500
C26—C27	1.373 (7)	C78—C79	1.373 (9)
C26—C31	1.383 (7)	C78—H78	0.9500
C27—C28	1.398 (8)	C79—C80	1.383 (8)
C27—H27	0.9500	C79—H79	0.9500
C28—C29	1.382 (8)	C80—H80	0.9500
C28—H28	0.9500	C81—C82	1.384 (7)
C29—C30	1.369 (8)	C81—C86	1.395 (7)
C29—H29	0.9500	C82—C83	1.398 (7)
C30—C31	1.384 (8)	C82—H82	0.9500
C30—H30	0.9500	C83—C84	1.368 (8)
C31—H31	0.9500	C83—H83	0.9500
C32—C37	1.393 (7)	C84—C85	1.374 (8)
C32—C33	1.401 (7)	C84—H84	0.9500
C33—C34	1.387 (8)	C85—C86	1.399 (7)
C33—H33	0.9500	C85—H85	0.9500
C34—C35	1.369 (9)	C86—H86	0.9500
			
P1—Cu1—P2	118.63 (5)	C36—C35—H35	119.9
P1—Cu1—S1	104.71 (6)	C35—C36—C37	119.6 (6)
P2—Cu1—S1	111.08 (6)	C35—C36—H36	120.2
P1—Cu1—I1	110.24 (4)	C37—C36—H36	120.2
P2—Cu1—I1	100.32 (4)	C32—C37—C36	121.2 (5)
S1—Cu1—I1	112.06 (4)	C32—C37—H37	119.4
P3—Cu2—P4	122.18 (5)	C36—C37—H37	119.4
P3—Cu2—S2	101.63 (5)	C43—C38—C39	118.9 (5)
P4—Cu2—S2	109.15 (5)	C43—C38—P2	116.9 (4)
P3—Cu2—I2	104.23 (4)	C39—C38—P2	124.0 (4)
P4—Cu2—I2	109.85 (4)	C38—C39—C40	118.7 (6)
S2—Cu2—I2	109.05 (4)	C38—C39—H39	120.6
C1—S1—Cu1	111.92 (19)	C40—C39—H39	120.6
C44—S2—Cu2	112.02 (19)	C41—C40—C39	121.5 (6)
C1—N1—H1A	120.0	C41—C40—H40	119.2
C1—N1—H1B	120.0	C39—C40—H40	119.2
H1A—N1—H1B	120.0	C42—C41—C40	119.0 (6)
C1—N2—C2	130.9 (5)	C42—C41—H41	120.5
C1—N2—H2A	114.6	C40—C41—H41	120.5
C2—N2—H2A	114.6	C41—C42—C43	120.3 (7)
C44—N3—H3A	120.0	C41—C42—H42	119.9
C44—N3—H3B	120.0	C43—C42—H42	119.9
H3A—N3—H3B	120.0	C42—C43—C38	121.6 (6)
C44—N4—C45	128.0 (4)	C42—C43—H43	119.2
C44—N4—H4A	116.0	C38—C43—H43	119.2
C45—N4—H4A	116.0	N4—C44—N3	119.9 (5)
C20—P1—C14	103.9 (2)	N4—C44—S2	120.4 (4)
C20—P1—C8	99.8 (2)	N3—C44—S2	119.7 (4)
C14—P1—C8	104.6 (2)	C46—C45—C50	120.6 (5)
C20—P1—Cu1	117.67 (19)	C46—C45—N4	118.2 (5)
C14—P1—Cu1	110.39 (17)	C50—C45—N4	121.0 (5)
C8—P1—Cu1	118.67 (17)	C45—C46—C47	120.2 (5)
C32—P2—C26	102.7 (2)	C45—C46—H46	119.9
C32—P2—C38	105.0 (3)	C47—C46—H46	119.9
C26—P2—C38	101.0 (2)	C48—C47—C46	119.9 (6)
C32—P2—Cu1	111.06 (17)	C48—C47—H47	120.1
C26—P2—Cu1	117.76 (19)	C46—C47—H47	120.1
C38—P2—Cu1	117.57 (18)	C49—C48—C47	119.9 (5)
C63—P3—C51	102.3 (2)	C49—C48—H48	120.1
C63—P3—C57	104.5 (2)	C47—C48—H48	120.1
C51—P3—C57	104.1 (2)	C48—C49—C50	120.3 (5)
C63—P3—Cu2	115.18 (17)	C48—C49—H49	119.8
C51—P3—Cu2	115.14 (17)	C50—C49—H49	119.8
C57—P3—Cu2	114.08 (17)	C45—C50—C49	119.1 (5)
C75—P4—C69	105.1 (2)	C45—C50—H50	120.5
C75—P4—C81	97.9 (2)	C49—C50—H50	120.5
C69—P4—C81	105.0 (2)	C52—C51—C56	117.1 (5)
C75—P4—Cu2	118.66 (19)	C52—C51—P3	118.1 (4)
C69—P4—Cu2	111.57 (17)	C56—C51—P3	124.8 (4)
C81—P4—Cu2	116.81 (17)	C51—C52—C53	121.5 (6)
N1—C1—N2	119.0 (5)	C51—C52—H52	119.2
N1—C1—S1	121.2 (4)	C53—C52—H52	119.2
N2—C1—S1	119.8 (4)	C54—C53—C52	120.5 (6)
C3—C2—C7	121.0 (5)	C54—C53—H53	119.8
C3—C2—N2	116.3 (5)	C52—C53—H53	119.8
C7—C2—N2	122.4 (5)	C53—C54—C55	119.1 (6)
C2—C3—C4	119.1 (5)	C53—C54—H54	120.5
C2—C3—H3	120.4	C55—C54—H54	120.5
C4—C3—H3	120.4	C54—C55—C56	120.6 (6)
C5—C4—C3	121.1 (6)	C54—C55—H55	119.7
C5—C4—H4	119.5	C56—C55—H55	119.7
C3—C4—H4	119.5	C51—C56—C55	121.0 (6)
C6—C5—C4	119.2 (5)	C51—C56—H56	119.5
C6—C5—H5	120.4	C55—C56—H56	119.5
C4—C5—H5	120.4	C58—C57—C62	118.1 (5)
C5—C6—C7	121.9 (6)	C58—C57—P3	118.2 (4)
C5—C6—H6	119.0	C62—C57—P3	123.6 (4)
C7—C6—H6	119.0	C59—C58—C57	120.8 (5)
C6—C7—C2	117.7 (6)	C59—C58—H58	119.6
C6—C7—H7	121.2	C57—C58—H58	119.6
C2—C7—H7	121.2	C60—C59—C58	120.5 (6)
C9—C8—C13	119.0 (5)	C60—C59—H59	119.7
C9—C8—P1	117.8 (4)	C58—C59—H59	119.7
C13—C8—P1	122.9 (4)	C59—C60—C61	119.5 (6)
C10—C9—C8	120.8 (6)	C59—C60—H60	120.2
C10—C9—H9	119.6	C61—C60—H60	120.2
C8—C9—H9	119.6	C60—C61—C62	121.0 (5)
C9—C10—C11	120.4 (6)	C60—C61—H61	119.5
C9—C10—H10	119.8	C62—C61—H61	119.5
C11—C10—H10	119.8	C61—C62—C57	120.0 (5)
C12—C11—C10	118.7 (6)	C61—C62—H62	120.0
C12—C11—H11	120.7	C57—C62—H62	120.0
C10—C11—H11	120.7	C64—C63—C68	118.7 (5)
C11—C12—C13	121.5 (6)	C64—C63—P3	123.5 (4)
C11—C12—H12	119.2	C68—C63—P3	117.7 (4)
C13—C12—H12	119.2	C65—C64—C63	120.2 (5)
C8—C13—C12	119.6 (5)	C65—C64—H64	119.9
C8—C13—H13	120.2	C63—C64—H64	119.9
C12—C13—H13	120.2	C66—C65—C64	120.1 (5)
C15—C14—C19	118.1 (5)	C66—C65—H65	120.0
C15—C14—P1	120.0 (4)	C64—C65—H65	120.0
C19—C14—P1	121.5 (4)	C67—C66—C65	120.3 (5)
C16—C15—C14	121.0 (5)	C67—C66—H66	119.9
C16—C15—H15	119.5	C65—C66—H66	119.9
C14—C15—H15	119.5	C68—C67—C66	119.5 (5)
C15—C16—C17	120.3 (5)	C68—C67—H67	120.2
C15—C16—H16	119.8	C66—C67—H67	120.2
C17—C16—H16	119.8	C67—C68—C63	121.2 (5)
C18—C17—C16	119.7 (5)	C67—C68—H68	119.4
C18—C17—H17	120.2	C63—C68—H68	119.4
C16—C17—H17	120.2	C74—C69—C70	118.2 (5)
C17—C18—C19	119.9 (5)	C74—C69—P4	118.2 (4)
C17—C18—H18	120.1	C70—C69—P4	123.1 (4)
C19—C18—H18	120.1	C71—C70—C69	120.4 (5)
C18—C19—C14	121.0 (5)	C71—C70—H70	119.8
C18—C19—H19	119.5	C69—C70—H70	119.8
C14—C19—H19	119.5	C70—C71—C72	120.9 (5)
C25—C20—C21	118.2 (5)	C70—C71—H71	119.5
C25—C20—P1	120.0 (4)	C72—C71—H71	119.5
C21—C20—P1	121.7 (4)	C71—C72—C73	119.4 (5)
C22—C21—C20	120.1 (5)	C71—C72—H72	120.3
C22—C21—H21	120.0	C73—C72—H72	120.3
C20—C21—H21	120.0	C72—C73—C74	119.9 (5)
C23—C22—C21	121.2 (5)	C72—C73—H73	120.1
C23—C22—H22	119.4	C74—C73—H73	120.1
C21—C22—H22	119.4	C73—C74—C69	121.2 (5)
C22—C23—C24	119.6 (5)	C73—C74—H74	119.4
C22—C23—H23	120.2	C69—C74—H74	119.4
C24—C23—H23	120.2	C76—C75—C80	118.3 (5)
C23—C24—C25	119.9 (6)	C76—C75—P4	119.9 (4)
C23—C24—H24	120.0	C80—C75—P4	121.6 (4)
C25—C24—H24	120.0	C75—C76—C77	119.7 (6)
C24—C25—C20	121.0 (5)	C75—C76—H76	120.1
C24—C25—H25	119.5	C77—C76—H76	120.1
C20—C25—H25	119.5	C78—C77—C76	120.8 (6)
C27—C26—C31	119.1 (5)	C78—C77—H77	119.6
C27—C26—P2	119.0 (4)	C76—C77—H77	119.6
C31—C26—P2	121.7 (4)	C77—C78—C79	120.3 (6)
C26—C27—C28	120.9 (6)	C77—C78—H78	119.9
C26—C27—H27	119.6	C79—C78—H78	119.9
C28—C27—H27	119.6	C78—C79—C80	120.1 (6)
C29—C28—C27	119.2 (6)	C78—C79—H79	120.0
C29—C28—H28	120.4	C80—C79—H79	120.0
C27—C28—H28	120.4	C79—C80—C75	120.8 (6)
C30—C29—C28	120.1 (6)	C79—C80—H80	119.6
C30—C29—H29	120.0	C75—C80—H80	119.6
C28—C29—H29	120.0	C82—C81—C86	119.1 (5)
C29—C30—C31	120.4 (6)	C82—C81—P4	124.6 (4)
C29—C30—H30	119.8	C86—C81—P4	116.1 (4)
C31—C30—H30	119.8	C81—C82—C83	119.8 (5)
C26—C31—C30	120.4 (5)	C81—C82—H82	120.1
C26—C31—H31	119.8	C83—C82—H82	120.1
C30—C31—H31	119.8	C84—C83—C82	120.8 (5)
C37—C32—C33	117.9 (5)	C84—C83—H83	119.6
C37—C32—P2	121.1 (4)	C82—C83—H83	119.6
C33—C32—P2	120.7 (4)	C83—C84—C85	120.3 (5)
C34—C33—C32	120.2 (6)	C83—C84—H84	119.9
C34—C33—H33	119.9	C85—C84—H84	119.9
C32—C33—H33	119.9	C84—C85—C86	119.6 (5)
C35—C34—C33	120.9 (6)	C84—C85—H85	120.2
C35—C34—H34	119.6	C86—C85—H85	120.2
C33—C34—H34	119.6	C81—C86—C85	120.4 (5)
C34—C35—C36	120.2 (6)	C81—C86—H86	119.8
C34—C35—H35	119.9	C85—C86—H86	119.8
			
P1—Cu1—S1—C1	−155.0 (2)	P2—C32—C33—C34	174.0 (5)
P2—Cu1—S1—C1	75.8 (2)	C32—C33—C34—C35	1.1 (10)
I1—Cu1—S1—C1	−35.5 (2)	C33—C34—C35—C36	−1.6 (10)
P3—Cu2—S2—C44	154.2 (2)	C34—C35—C36—C37	0.9 (10)
P4—Cu2—S2—C44	−75.5 (2)	C33—C32—C37—C36	−0.9 (8)
I2—Cu2—S2—C44	44.5 (2)	P2—C32—C37—C36	−174.7 (5)
P2—Cu1—P1—C20	−49.5 (2)	C35—C36—C37—C32	0.4 (9)
S1—Cu1—P1—C20	−174.04 (19)	C32—P2—C38—C43	160.6 (5)
I1—Cu1—P1—C20	65.3 (2)	C26—P2—C38—C43	−92.9 (5)
P2—Cu1—P1—C14	69.5 (2)	Cu1—P2—C38—C43	36.6 (5)
S1—Cu1—P1—C14	−55.1 (2)	C32—P2—C38—C39	−25.2 (6)
I1—Cu1—P1—C14	−175.77 (19)	C26—P2—C38—C39	81.3 (6)
P2—Cu1—P1—C8	−169.8 (2)	Cu1—P2—C38—C39	−149.2 (5)
S1—Cu1—P1—C8	65.6 (2)	C43—C38—C39—C40	1.0 (9)
I1—Cu1—P1—C8	−55.1 (2)	P2—C38—C39—C40	−173.1 (5)
P1—Cu1—P2—C32	52.3 (2)	C38—C39—C40—C41	−1.9 (10)
S1—Cu1—P2—C32	173.66 (19)	C39—C40—C41—C42	1.1 (11)
I1—Cu1—P2—C32	−67.70 (19)	C40—C41—C42—C43	0.6 (11)
P1—Cu1—P2—C26	−65.6 (2)	C41—C42—C43—C38	−1.5 (11)
S1—Cu1—P2—C26	55.7 (2)	C39—C38—C43—C42	0.6 (9)
I1—Cu1—P2—C26	174.36 (19)	P2—C38—C43—C42	175.1 (5)
P1—Cu1—P2—C38	173.2 (2)	C45—N4—C44—N3	−2.6 (8)
S1—Cu1—P2—C38	−65.5 (2)	C45—N4—C44—S2	177.4 (4)
I1—Cu1—P2—C38	53.2 (2)	Cu2—S2—C44—N4	−13.3 (5)
P4—Cu2—P3—C63	51.5 (2)	Cu2—S2—C44—N3	166.7 (4)
S2—Cu2—P3—C63	173.1 (2)	C44—N4—C45—C46	−125.8 (6)
I2—Cu2—P3—C63	−73.5 (2)	C44—N4—C45—C50	57.6 (8)
P4—Cu2—P3—C51	170.3 (2)	C50—C45—C46—C47	−0.4 (8)
S2—Cu2—P3—C51	−68.1 (2)	N4—C45—C46—C47	−177.0 (5)
I2—Cu2—P3—C51	45.3 (2)	C45—C46—C47—C48	0.7 (9)
P4—Cu2—P3—C57	−69.39 (19)	C46—C47—C48—C49	−0.6 (9)
S2—Cu2—P3—C57	52.28 (19)	C47—C48—C49—C50	0.3 (9)
I2—Cu2—P3—C57	165.61 (18)	C46—C45—C50—C49	0.1 (8)
P3—Cu2—P4—C75	−48.3 (2)	N4—C45—C50—C49	176.6 (5)
S2—Cu2—P4—C75	−166.35 (19)	C48—C49—C50—C45	0.0 (9)
I2—Cu2—P4—C75	74.13 (19)	C63—P3—C51—C52	176.1 (5)
P3—Cu2—P4—C69	74.04 (19)	C57—P3—C51—C52	−75.3 (5)
S2—Cu2—P4—C69	−44.03 (18)	Cu2—P3—C51—C52	50.3 (5)
I2—Cu2—P4—C69	−163.55 (17)	C63—P3—C51—C56	−4.5 (6)
P3—Cu2—P4—C81	−165.18 (19)	C57—P3—C51—C56	104.1 (5)
S2—Cu2—P4—C81	76.8 (2)	Cu2—P3—C51—C56	−130.2 (5)
I2—Cu2—P4—C81	−42.8 (2)	C56—C51—C52—C53	−0.2 (10)
C2—N2—C1—N1	0.8 (9)	P3—C51—C52—C53	179.2 (5)
C2—N2—C1—S1	−179.7 (5)	C51—C52—C53—C54	4.0 (11)
Cu1—S1—C1—N1	−152.6 (4)	C52—C53—C54—C55	−4.9 (11)
Cu1—S1—C1—N2	28.1 (5)	C53—C54—C55—C56	2.1 (11)
C1—N2—C2—C3	139.7 (6)	C52—C51—C56—C55	−2.6 (10)
C1—N2—C2—C7	−47.4 (9)	P3—C51—C56—C55	178.0 (5)
C7—C2—C3—C4	−1.1 (9)	C54—C55—C56—C51	1.7 (11)
N2—C2—C3—C4	171.9 (5)	C63—P3—C57—C58	−98.3 (4)
C2—C3—C4—C5	1.1 (9)	C51—P3—C57—C58	154.7 (4)
C3—C4—C5—C6	0.3 (9)	Cu2—P3—C57—C58	28.3 (4)
C4—C5—C6—C7	−1.8 (9)	C63—P3—C57—C62	81.5 (5)
C5—C6—C7—C2	1.8 (9)	C51—P3—C57—C62	−25.6 (5)
C3—C2—C7—C6	−0.3 (9)	Cu2—P3—C57—C62	−151.9 (4)
N2—C2—C7—C6	−172.9 (5)	C62—C57—C58—C59	−2.2 (8)
C20—P1—C8—C9	−99.4 (5)	P3—C57—C58—C59	177.6 (4)
C14—P1—C8—C9	153.3 (4)	C57—C58—C59—C60	3.0 (8)
Cu1—P1—C8—C9	29.8 (5)	C58—C59—C60—C61	−1.4 (8)
C20—P1—C8—C13	74.6 (5)	C59—C60—C61—C62	−1.0 (8)
C14—P1—C8—C13	−32.7 (5)	C60—C61—C62—C57	1.8 (8)
Cu1—P1—C8—C13	−156.3 (4)	C58—C57—C62—C61	−0.2 (7)
C13—C8—C9—C10	−2.4 (9)	P3—C57—C62—C61	−180.0 (4)
P1—C8—C9—C10	171.8 (5)	C51—P3—C63—C64	117.5 (5)
C8—C9—C10—C11	1.4 (10)	C57—P3—C63—C64	9.2 (5)
C9—C10—C11—C12	0.3 (10)	Cu2—P3—C63—C64	−116.8 (4)
C10—C11—C12—C13	−0.9 (9)	C51—P3—C63—C68	−66.1 (5)
C9—C8—C13—C12	1.8 (8)	C57—P3—C63—C68	−174.4 (4)
P1—C8—C13—C12	−172.1 (4)	Cu2—P3—C63—C68	59.6 (5)
C11—C12—C13—C8	−0.2 (9)	C68—C63—C64—C65	−1.1 (8)
C20—P1—C14—C15	−153.0 (4)	P3—C63—C64—C65	175.3 (4)
C8—P1—C14—C15	−48.8 (5)	C63—C64—C65—C66	1.4 (9)
Cu1—P1—C14—C15	80.0 (4)	C64—C65—C66—C67	−0.4 (9)
C20—P1—C14—C19	33.9 (5)	C65—C66—C67—C68	−1.0 (9)
C8—P1—C14—C19	138.1 (4)	C66—C67—C68—C63	1.3 (9)
Cu1—P1—C14—C19	−93.2 (4)	C64—C63—C68—C67	−0.3 (8)
C19—C14—C15—C16	0.1 (8)	P3—C63—C68—C67	−176.8 (4)
P1—C14—C15—C16	−173.3 (4)	C75—P4—C69—C74	−173.9 (4)
C14—C15—C16—C17	−0.6 (8)	C81—P4—C69—C74	−71.1 (4)
C15—C16—C17—C18	0.6 (8)	Cu2—P4—C69—C74	56.3 (4)
C16—C17—C18—C19	−0.2 (8)	C75—P4—C69—C70	14.3 (5)
C17—C18—C19—C14	−0.3 (8)	C81—P4—C69—C70	117.1 (4)
C15—C14—C19—C18	0.3 (7)	Cu2—P4—C69—C70	−115.5 (4)
P1—C14—C19—C18	173.6 (4)	C74—C69—C70—C71	−0.7 (8)
C14—P1—C20—C25	−146.2 (4)	P4—C69—C70—C71	171.1 (4)
C8—P1—C20—C25	106.0 (5)	C69—C70—C71—C72	−0.2 (9)
Cu1—P1—C20—C25	−23.8 (5)	C70—C71—C72—C73	1.2 (9)
C14—P1—C20—C21	36.4 (5)	C71—C72—C73—C74	−1.2 (8)
C8—P1—C20—C21	−71.5 (5)	C72—C73—C74—C69	0.2 (8)
Cu1—P1—C20—C21	158.8 (4)	C70—C69—C74—C73	0.7 (8)
C25—C20—C21—C22	0.9 (8)	P4—C69—C74—C73	−171.5 (4)
P1—C20—C21—C22	178.4 (4)	C69—P4—C75—C76	−130.9 (4)
C20—C21—C22—C23	−1.9 (8)	C81—P4—C75—C76	121.1 (4)
C21—C22—C23—C24	1.7 (9)	Cu2—P4—C75—C76	−5.4 (5)
C22—C23—C24—C25	−0.5 (9)	C69—P4—C75—C80	54.2 (5)
C23—C24—C25—C20	−0.4 (9)	C81—P4—C75—C80	−53.8 (5)
C21—C20—C25—C24	0.2 (8)	Cu2—P4—C75—C80	179.7 (4)
P1—C20—C25—C24	−177.3 (4)	C80—C75—C76—C77	−1.0 (8)
C32—P2—C26—C27	−115.7 (4)	P4—C75—C76—C77	−176.1 (4)
C38—P2—C26—C27	136.1 (4)	C75—C76—C77—C78	0.2 (9)
Cu1—P2—C26—C27	6.7 (5)	C76—C77—C78—C79	0.0 (10)
C32—P2—C26—C31	59.6 (5)	C77—C78—C79—C80	0.5 (10)
C38—P2—C26—C31	−48.7 (5)	C78—C79—C80—C75	−1.4 (9)
Cu1—P2—C26—C31	−178.1 (4)	C76—C75—C80—C79	1.6 (8)
C31—C26—C27—C28	1.2 (8)	P4—C75—C80—C79	176.6 (4)
P2—C26—C27—C28	176.6 (4)	C75—P4—C81—C82	107.0 (5)
C26—C27—C28—C29	−1.1 (9)	C69—P4—C81—C82	−1.0 (5)
C27—C28—C29—C30	1.4 (9)	Cu2—P4—C81—C82	−125.2 (4)
C28—C29—C30—C31	−1.7 (9)	C75—P4—C81—C86	−68.4 (4)
C27—C26—C31—C30	−1.5 (8)	C69—P4—C81—C86	−176.4 (4)
P2—C26—C31—C30	−176.8 (4)	Cu2—P4—C81—C86	59.4 (4)
C29—C30—C31—C26	1.8 (9)	C86—C81—C82—C83	0.6 (8)
C26—P2—C32—C37	−156.6 (4)	P4—C81—C82—C83	−174.7 (4)
C38—P2—C32—C37	−51.4 (5)	C81—C82—C83—C84	0.1 (8)
Cu1—P2—C32—C37	76.7 (5)	C82—C83—C84—C85	−0.1 (8)
C26—P2—C32—C33	29.8 (5)	C83—C84—C85—C86	−0.4 (8)
C38—P2—C32—C33	135.0 (4)	C82—C81—C86—C85	−1.1 (8)
Cu1—P2—C32—C33	−97.0 (4)	P4—C81—C86—C85	174.6 (4)
C37—C32—C33—C34	0.1 (8)	C84—C85—C86—C81	1.1 (8)

### Hydrogen-bond geometry (Å, °)

**Table:**

*D*—H···*A*	*D*—H	H···*A*	*D*···*A*	*D*—H···*A*
N1—H1B···S2^i^	0.88	2.58	3.455 (5)	173
N3—H3B···S1^ii^	0.88	2.63	3.396 (5)	146
N2—H2A···I1	0.88	2.65	3.511 (5)	166
N4—H4A···I2	0.88	2.71	3.567 (4)	165

Symmetry codes: (i) *x*−1, *y*+1, *z*; (ii) *x*+1, *y*−1, *z*.
